# At the nexus of science, engineering, and medicine: Pasteur's quadrant reconsidered

**DOI:** 10.1093/pnasnexus/pgac092

**Published:** 2022-06-23

**Authors:** Roderic I Pettigrew, John P Cooke

**Affiliations:** School of Engineering Medicine, Texas A&M University, Houston, TX, USA; Department of Cardiovascular Sciences, Houston Methodist Research Institute, Houston, TX, USA; School of Engineering Medicine, Texas A&M University, Houston, TX, USA; Department of Cardiovascular Sciences, Houston Methodist Research Institute, Houston, TX, USA

## Abstract

There has been a sea change in the scientific world, advanced even more rapidly by the recent compounded public crises. Accelerated discovery, and impact from such discoveries have come from convergence approaches across disciplines, sectors, institutions, and the multiple communities seeking the common goal of innovations that transform. The classic simultaneous pursuit of fundamental understanding and application has been termed  Pasteur's quadrant, where use-inspired basic research occurs. In the classic schematic developed by Donald Stokes, three quadrants  represent research approaches using a 2D plane in which the vertical dimension represents the quest for understanding (basic research) and the horizontal dimension represents the consideration of use (applied research). The three outer quadrants are Bohr's (pure basic research), Edison's (pure applied research), and Pasteur's (use-inspired  basic research). Viewing each of these axes as a continuum, we label the previously unnamed but contributory cell as the Innominate quadrant, where a nonzero amount of discovery and applied research also has value in generating scientific tools, novel processes or products that inform the other quadrants. More importantly, a reimagined Pasteur's quadrant schema shows a third dimension of Transformations over Time, occurring through a continuous fluid interchange among the quadrants.  Transformative innovations may originate from any single quadrant.  While work in Pasteur's quadrant has been shown to be highly productive, a dynamic fluid interchange among the quadrants is often involved and generates transformative advances at a faster rate. This should inform how we fund science, engineering, and medicine and educate the next generation of innovators.

## The Next Generation of Innovation

### The dawning of a new age of transformative advances

The far-reaching consequences of compounded public crises resulting from and exposed by the recent pandemic have had one overarching positive effect. The pandemic accelerated convergence among science, engineering, and medicine. A logical movement that began organically many years ago shifted into overdrive and taught us operative lessons that if followed, could reap substantial scientific benefit for all generations to follow. And these are lessons not to be forgotten as some of the most pressing concerns of the pandemic fade.

Indeed, there has been a sea change in the scientific world. Accelerated discovery, and impact from such discoveries have come from convergence approaches across disciplines, sectors, institutions, and the multiple communities seeking the common goal of innovations that transform (1, 2). Our research and development world is exponentially expanding, aided by hyperconnectivity with shared learning across the globe utilizing convergence principles. In this approach, multiple science and engineering fields are merged to create new insights and disciplines with the potential for breakthroughs in problem solving (1,3). Most recently, this includes artificial intelligence with deep learning to extract information beyond simple human observation, multiomics single cell profiling with computational algorithms to derive new insights from these massive datasets, computational modeling for efficient and intelligent approaches to drug design insights, mixed reality visualization to enhance information utility, super resolution imaging of natural processes to discover unknown phenomena, smart platforms for diagnostics, and therapeutics that mimic our own physiology, and data science with holistic systems approaches for greater utility and efficiency in disease prevention, prediction, detection, and treatment. A new day has dawned through the logic and power of convergence. This has been demonstratively illustrated by the converged interdisciplinary effort that has helped to counter the highly transmissible SARS-CoV-2 virus and its variants.

Generally, the scientific community has capitalized on its relatively recent history of interdisciplinary science inclusive of technological innovations, which have generated new fundamental understandings of human biology, physiology, health, and disease. We have also leveraged the growth in open science to further accelerate discovery. We are poised to facilitate an even deeper integration of disciplines with a convergence that is adaptive to deliver practical and accessible solutions to major problems in our contemporary society. A review of the details of these advances, however, shows that the path to innovations that have transformed lives did not occur as a result of either pure basic or pure applied research. Rather more often transformative advances result from a dynamic process of informed interchange among these domains.. These advances may start as curiosity driven investigations, seeking fundamental knowledge (e.g. can atoms be stimulated to emit coherent light) and later be inspired by a practical need (e.g. light beams to etch micro cuts in polymers; laser as a scalpel), or can begin with tool development (e.g. imaging below the light diffraction limit) and lead to fundamental knowledge (e.g. the 4D embryological development pattern of neurons) (4). Transformative advances typically occur at the intersection of discovery and invention, but the path can proceed iteratively in either direction. There is a natural need for “freedom to pivot quickly between science and invention” as the purpose, need, or opportunity arises (5).

The success and impact of this type of dynamic convergence has been quite remarkable: 4D noninvasive imaging of human pathophysiology, gene editing by adapting bacterial molecular defense mechanisms, and immunoengineering with mRNA modulation of the immune system in humans. This illustrates what investments in high level basic discovery and open sharing of data with use-inspired technological innovations can yield. Additional emerging investigations at the nexus are numerous. Examples include multiomic single cell analyses to generate a neuronal cell atlas and signaling interactions, maps of human thought and emotions to provide insights into language, communication, and behavioral health, an understanding of the molecular basis of drug addiction, and restoration of neurohormonal regulation for cardiovascular health.

These are marvelous indeed, but the call for convergence to meet our great challenges of today and tomorrow is clear and compelling. There are, for example, pressing opportunities to regenerate normal cognitive function after brain injury, to reverse major emotional disorders, to halt the progression of neurodegenerative disorders, to normalize metabolism and eliminate diabetes, to restore cardiovascular homeostasis and eliminate heart attacks, and to generate effective vaccines against any pathogen or cancer. These are all within the reach of our imaginations and each challenge calls for this nexus.

Indeed, the deep challenge of social inequities in healthcare access, delivery, and even training stalks our global society and challenges our best and brightest minds, institutions, and policy makers. The convergence of science—including behavioral and social science, the humanities, engineering, and medicine are needed to develop our most efficient and effective solutions to this array of vexing problems. This nexus-based paradigm offers the best and most strategic pathway into the 22nd century that will achieve a greatly improved human condition.

### The nature of transformational advances requires a re-evaluation of Pasteur's quadrant

**Fig. 1.**
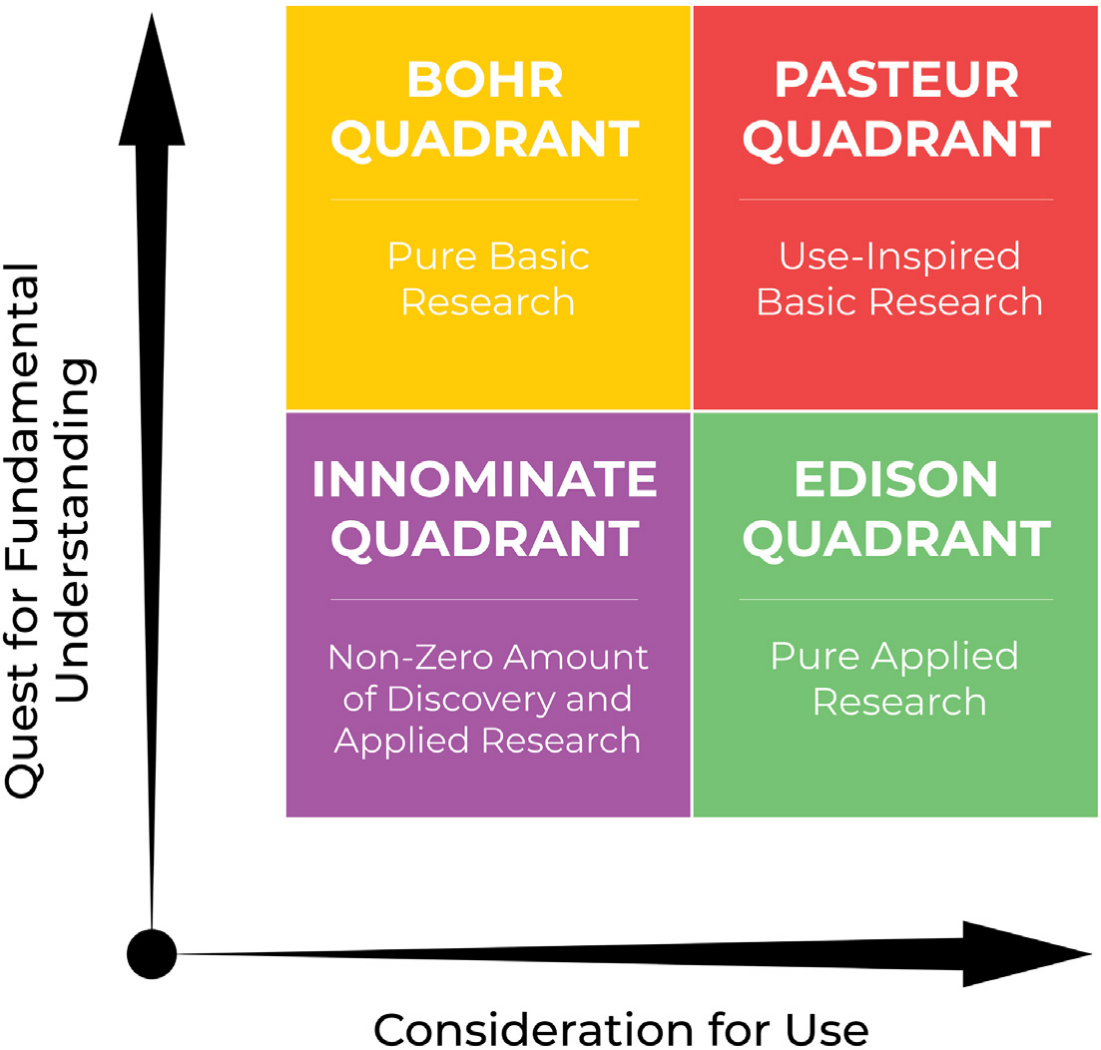
Pasteur's quadrant schematic as developed by Donald Stokes, modified to show a continuum of *Consideration for Use* and *Quest for Fundamental Understanding* and to include a name for the quadrant at the lower left. The classic Stokes Diagram represented research approaches using a 2D plane in which the vertical dimension, for heuristic purposes, had binary Yes–No choices in the quest for understanding (basic research), whereas the horizontal dimension had a binary choice in the consideration of use (applied research). The three outer quadrants are Bohr's (pure basic research), Edison's (pure applied research), and Pasteur's (use-inspired research). We label the previously unnamed lower left cell under the continuous axes, the Innominate quadrant, where a nonzero amount of discovery and applied research also has value in generating scientific tools, novel processes, platforms, or products that inform and facilitate the other quadrants.

In his book *Pasteur's Quadrant—Basic Science and Technological Innovation*, Donald Stokes describes an orthogonal relationship between research that is inspired by the quest for fundamental understanding (basic research) and research inspired by a consideration of use (applied research) (6). These axes establish a 2D four quadrant grid in which the upper left quadrant represents fundamental research without consideration of use and is typified by the work of physicist Niels Bohr, who posited the quantum mechanical model of the atom and electron orbitals. The lower right quadrant is use-inspired research without a quest for fundamental understanding, or pure applied research, and is typified by the work of Thomas Edison and his invention of the light bulb. In the upper right quadrant is an approach that simultaneously seeks both fundamental understanding and  application, such as that exemplified by Louis Pasteur's microbiology work that led to vaccines. In this four-quadrant grid construct, Pasteur's quadrant has been previously posited as the most desirable for research activities that will deliver transformative innovations (Fig 1) (7). Nonetheless, Stokes does note that interchange among research in these quadrants may at times occur and consequentially realize scientific progress.

Indeed, the growing and varied experiences with convergence research suggests a need for greater appreciation of this dynamic process that is most effective in producing transformative innovations. In this reconsidered conceptual framework, we emphasize that a fluid and continuous interchange among the quadrants is most likely to advance life over time, and thus should be encouraged and funded. Indeed, the work and discoveries of Pasteur himself were at different times driven by a quest for understanding (microbes and fermentation) and later use or application (inhibiting infection through an engineered vaccine). Even with Edison, there was a need to understand the flow of electrons in filaments and specific element-based wire impedance in order to effect a long lasting light bulb as the early bulbs would last only ∼ 15 minutes. And Bohr's model of the atom gave rise to intelligent use of gases for fluorescent lighting and eventually to transistors.

There is a fourth quadrant in the Stokes diagram that has been largely overlooked by history, or dismissively labeled as tinkering. A total of 25 years ago, Stokes described this quadrant as including “research that systematically explores particular phenomena without having in view either general explanatory objectives or any applied use to which the results will be put …studies in the this quadrant can be important precursors of research in Bohr's quadrant …as well as of research in Edison's quadrant. ” Stokes provided the example of Peterson's work, which systematically described avian plumage, as subsequently being useful to birdwatchers. He deferred from labeling this Peterson's quadrant, however, because of the work's limited reach. Whereas such research falls into this quadrant, so too might the acquisition or cataloguing of existing knowledge on a range of topics, or early laboratory studies or development of new platform technologies which require a nonzero amount of discovery and applied research. In a broad sense, this includes acquisition of information on a topic or topics that serve as a precursor to work in the other quadrants. A modern and dramatic example is the development of the World Wide Web, which is a curation of information that can be easily shared through a searchable linking system. We propose that the www is a modern exemplar of this fourth quadrant and underscores its value. One might also place in this quadrant new technologies that lead to discovery or invention. An example is photoactivatable proteins that underpin the development of super resolution microscopy for which the Nobel Prize was awarded in 2014. Accordingly, following Stokes’ description and noting that his considerations did not result in an exemplar person's name, we have labeled the lower left cell in the new diagram the Innominate quadrant. This quadrant, in our view, is the domain of a range of research elements inclusive of the problem-based evolution of tools, model systems, heuristic approaches, or conceptual frameworks. At higher values of the coordinates within this quadrant reside foundational developments for transformation such as photoactivatable proteins or platforms such as the World-Wide Web. All of these speak to the importance of this quadrant. They delineate its value in our modern culture of converged research and explain how work in this quadrant, as a precursor to, or platform for invention or discovery also contributes to the fluid dynamic among all the quadrants.

Progressive interchange among all four quadrants is particularly true when one considers each of the axes as a continuum and not simply as a dichotomous yes or no choice as in the single Stokes diagram that is often referenced. In fact, Stokes describes a spectrum of research in both directions. But in his 2D diagram, he created a binary choice along each axis for “heuristic” reasons. The observation that there is a range of research activities along each axis and that there may be continuous interchanges among areas defined by the 2D classification, was also described by Stokes.

Perhaps even more evident today, modern science and technological innovations now arise from a more greenfield approach of fluid interchange and synergy among ideas flowing in and out of these quadrants at various points in time. Going beyond the often-referenced insular quadrants and essentially operating across this 2D grid of quadrants over time is a compelling paradigm. It is an approach that is most likely to yield transformations that improve lives.

### A new diagram and expanded implications

**Fig. 2.**
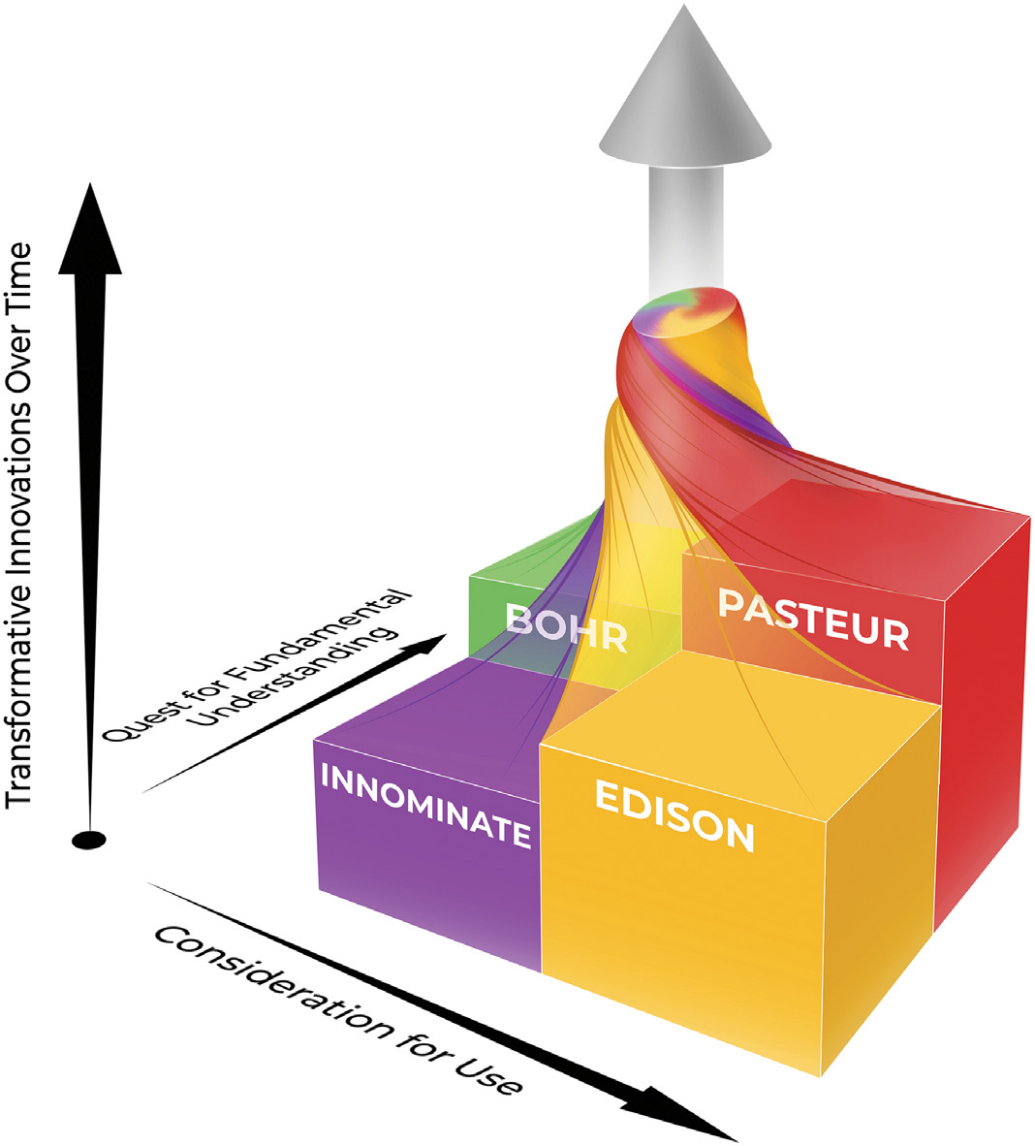
Reimagined Pasteur's quadrant diagram shows a third dimension of *Transformations over Time*, occurring through a continuous fluid interchange among the quadrants. Adding the third dimension illustrates that transformative innovations may originate from any single quadrant. However, a dynamic fluid interchange among the quadrants is often involved and generates transformative advances at a faster rate.

To better illustrate this concept, we expand the Stokes diagrams further by adding a third dimension, where the *z*-axis becomes transformational advances over time (Fig 2). Whereas transformational advances can occur in each quadrant, individuals or groups that can navigate freely among the quadrants can generate transformational advances at a faster rate. The swirling spirals with different starting points across the quadrants reflect the fact that different paths can be traveled by an investigator or group of collaborators to generate transformational advances. In some cases, the journey may begin with a serendipitous discovery that, to the convergent mind, suggests a potential use (moving from Bohr's, through Edison's, and thus to Pasteur's quadrant). However, this is not the exclusive pathway for transformational advances. For example, the refinement of a scientific tool may lead to its application in discovery (moving from the Innominate quadrant to Edison's or Bohr's quadrant). Indeed, many fundamental scientific discoveries were preceded by the refinement of a tool that permitted new observations and insights (e.g. Leeuwenhoek's fabrication of the first microscope permitted Pasteur’ s advances). Alternatively, the results of applied research may result in a hypothesis-generating advance that stimulates pure discovery (moving from Edison's to Bohr's quadrant).

To be sure, not all individuals are equipped nor inclined to navigate these quadrants individually, thus the need for convergence through interdisciplinary or transdisciplinary teams of collaborative investigators. Indeed, realization of innovations that transform in our contemporary society must also include social, behavioral, and use considerations. A new technology that is neither available nor inclusive of cultural humility may not reach all persons or have the intended impact if not embraced (e.g. scientifically valid new vaccinations and preventive measures not universally employed). The broader view of convergence, therefore, includes all natural, social and behavioral sciences, the arts, humanities, and engineering. Specifically, the social sciences may inform us on how to address attitudinal, behavioral, cultural, and sociopolitical barriers to improving the human condition through science and technology. Nonetheless, the central theme of a fluid interchange over time among research conducted in these reimagined quadrants, where each may be infused with these varied disciplines, still holds. It is, therefore, important to realize and support all quadrant efforts where appropriate because of the dynamic and intertwined process among them that leads to transformative innovations. Historically, Pasteur's quadrant has been touted because, it is converged. And indeed this approach (use-inspired basic research) among the individual quadrants produces the greatest level of transformations over time (Fig. 2). Yet, both researchers and funders should realize these are not insular or static domains. Rather continuous innovation results from movement into and out of these quadrants, including Pasteur's. The message of the 3D diagram, thus also encourages the creation of funding and research support policies to facilitate and accelerate this interchange dynamic. A recent example is the National Heart Lung and Blood Institute's Catalyze program which “facilitates the transition of basic science discoveries into viable diagnostic and therapeutic candidates cleared for human testing.” This program supports the translation of academic research by providing resources that, in our scheme, fit into the Innominate quadrant.

As this last example suggest, such an integration will require more effective and open collaboration among companies and universities, inclusive of work in the Innominate quadrant. Recognizing the importance of this quadrant for explorations in advancing university technology may inspire academic leadership to fund such work, and to provide the necessary facilities and mentorship. Industry can provide seed funds and guidance for the improvement of technologies arising from fundamental research in the university. An excellent example of an heuristic approach through a university–industry collaboration is the SPARK program at Stanford University, which has been very successful in commercializing intellectual property and spawning biotech startups. This program has now been replicated at many universities world-wide. Federal and Non-Governmental Organization funding of such initiatives will further augment and accelerate the translation of university technologies.

The importance of convergence in accelerating transformational advances is clear (7). That it can occur from a variety of paths over time resulting in transformations arising from any combination of quadrants is to be appreciated and supported. The graphic in Fig. 2 is also intended to illustrate this perspective with the quadrant's spirals intertwined and rising along the axis of transformational advances over time. The third axis also has import where there is a “consideration of urgency” characterizing research motivated by a pandemic or global challenge. Additionally, the vertical axis could also reflect “consideration of social value.” Each of these are implicit in the term “Transformational Advances over Time” when considered broadly where universal value is sought.

We submit that our conceptual framework represents the dynamic nature of scientific convergence, even when it occurs in the same mind, and that this fluid process is to be encouraged to meet our great global challenges. Indeed, we do need deep innovation and fundamental discovery where the focus is scientific truth seeking. We also need to translate those discoveries into advances for health. The integration of fundamental discovery and innovative applications will accelerate the work in all quadrants in an unending fluid interchange. It is this fluid interchange that is most productive.

## Lessons from Three Historic Examples

### From laser to lasik—the 20 year journey

Over 50 million people each year undergo a vision correction procedure in which a laser is carefully used to precisely deliver scar free radial cuts in the cornea. This changes the shape of the cornea and its focal length, which is adjusted to fall on the retina and sharpen vision. A key feature of this procedure is the use of monochromatic laser light at a precise wavelength or energy to cause cuts without excess heating that would produce scar tissue.

The laser was invented in the early 1960s with its inventors Charles Townes, Aleksandr Prokhorov, and Nicolai Basov being awarded the Nobel Prize in 1964. Its varied applications in scientific investigation, medicine, telecommunications, commerce, and entertainment are considerable. However, it was not until Rangaswamy Srinivasan and colleagues at IBM observed in the 1980s that far ultraviolet laser light could etch polymers cleanly, that the potential for precise tissue incisions was conceived (8). The quantum of energy delivered by the far UV laser is just enough to break peptide bonds without residual heating. The beauty of this effect is no residual burns or “collateral damage” as they described it. Srinivasan, struck by a moment of inspiration at his Thanksgiving dinner table, tested this concept on leftover turkey to evaluate its potential utility in tissue. The submicron laser cuts were indeed clean without burns. It was a few years later, that presentation of this discovery at a medical meeting inspired ophthalmologists from the Columbia University to test this far UV laser for photorefractive corneal surgery, leading to the first vision corrective surgery in a sighted human in 1988 (9).

This history illustrates the road to a transformative innovation involves, at different times, work in each of the four quadrants. Going forward, it is important to note, that had there been greater convergence earlier, laser vision correction procedures may have occurred much sooner. This points to the importance of the pursuit of convergence in the *design and funding* of our education, research, and translational activities (7).

### A “See” change in diagnostic medicine—3D human imaging

CT, PET, and MRI each generate 3D images of the human body. At the turn of the century, the development of 3D imaging was ranked in a national survey of physicians as the most important advance in medicine over the prior three decades (10). The development of MRI is an example of use-inspired research that subsequently also led to fundamental discovery. The development of MRI relied on the discovery of spatial encoding of radio signals produced by the precession of hydrogen nuclei of molecules placed in a magnetic field gradient. The initial application of this discovery was to generate a tool that could be applied to noninvasively visualize human anatomy and pathology in 3D, and to characterize any tissue differences revealed by the fundamental parameters of nuclear magnetic resonance. Subsequently, this led to more advanced use-inspired basic research such as neural fiber tractography and functional imaging to define and study the human connectome.

Indeed, the history of MRI development, application, and fundamental discovery in a continuous and ongoing cycle, is exemplary. One example is the remarkable progress in probing the mysteries of neuroscience and the brain. The NIH's BRAIN initiative (Brain Research through Advancing Innovative Neurotechnologies) exemplifies active convergence research evolving over time, continuously moving among the four quadrants in our reimagined Stokes model (11).

Indeed the BRAIN initiative has leveraged teams of our best and brightest minds from multiple disciplines to think big, aim high, and reach far beyond previous boundaries in human neuroscience. Research teams comprised of MRI physicist, electrical engineers,  cell and molecular neuroscientists, neuroradiologists,  neurologists, and computer scientists have worked  to gain both fundamental understanding and use-inspired technologies. Advances now include visualization of neural pathways during tumor surgery to avoid a neurologic deficit, an understanding that each of us has a unique connectome “fingerprint” that is predictive of some cognitive function features, and even visualization of neuro-activation patterns specific to emotions or feelings. Even the geometric construct of the brain's neuronal pathways with orthogonality at points of fiber crossings is a new fundamental discovery that will require additional investigations to truly understand its functional value  (12).

These basic discoveries and their use have led to more basic questions, sparking a new use-inspired invention. Scientists at MGH, supported by the NIH, are developing the first Human Dynamic Neurochemical Connectome. It will combine highest resolution MRI with a new PET sphere having a tenfold increased sensitivity (13). This device should provide unprecedented temporal and spatial resolution of neurochemical processes in the brain to enable dynamic PET imaging of neurotransmission, neuromodulation, and previously unstudied dynamic molecular events.  Many additional basic and practical payoffs await. These include assessing microcircuits and their interplay with hemodynamics, deciphering the blood–brain barrier construct and function, defining cellular metabolism and gene expression, investigating non-neuronal (glial) structure and function, measuring real-time drug distribution, and discovering ion channel dynamics in vivo (11).

### The emerging revolution in mRNA therapeutics

The life-saving generation of mRNA vaccines against the global affliction of SARS CoV-2 is just the beginning of a therapeutic revolution that will transform patient care. The mRNA technology can be imagined as a form of biological software, where one can rapidly write the code for any therapeutic protein or antigen. Within 24 hours of the release of the genomic sequence of SARS-CoV-2 on 2020 January 10, the RNA construct encoding the stabilized spike protein was generated, and within 5 days, cGMP production of the mRNA vaccine was initiated in parallel with preclinical evaluation. This led to a first-in-human phase I clinical trial of the Moderna vaccine 66 days after the viral sequence was released (14). The rapid development of mRNA therapeutics is reflected by the breadth of the product lines, and the high valuations of the relatively new companies that are currently leading the field. As we have observed, the pressure of the pandemic substantially accelerated the approval of the first mRNA therapeutics, a lesson that may inform the intentional stimulation of future transformational advances.

The question then arises as to why has it taken so long for mRNA therapeutics to emerge? After all, mRNA was discovered in 1961, and its first application as a therapeutic in preclinical studies was in 1992, when mRNA encoding vasopressin was used successfully to treat diabetes insipidus in a rodent model (15, 16). The delay from discovery to application lay in the hurdles that needed to be overcome for mRNA to be useful clinically. As described below, the development of the mRNA vaccines required contributions from all four quadrants of discovery and applied research. We posit that it took three decades to make this transformational advance in part because current funding and training efforts have not intentionally encouraged navigation through the four quadrants.

A major hurdle to RNA therapeutics was the delivery of negatively charged RNA across a hydrophobic cytoplasmic membrane, initially solved using cationic lipofection methodology (15, 16). However, these methods caused cellular and systemic toxicity, a problem which was ultimately overcome by the development of lipid nanoparticles (LNPs) comprised of ionizable lipids. Such LNPs traverse the cell membrane, and in the acidic environment of the lysosome, release their RNA cargo into the cytoplasm. The development of these LNPs required a fundamental understanding of the biophysics of cell membranes (Bohr's quadrant) and their interactions with LNPs, but it also required an empirical approach (Innominate quadrant) to optimize the lipid composition of the LNPs. This then became an effective vaccine delivery vehicle (Edison's quadrant).

A major obstacle to mRNA therapeutics was the inflammatory response to synthetic mRNA, which caused cell toxicity and/or impaired translation into a therapeutic protein. A seminal insight that advanced the field was the finding that in vitro transcribed (IVT) mRNA had the adverse effect of generating excessive inflammatory cytokines. This effect was due to the activation of innate immune signaling through pattern recognition receptors (PRRs) such as Toll-like receptors. The recognition that endogenous tRNAs were enriched in modified nucleosides and did not activate inflammatory signaling, stimulated the use of modified nucleosides in IVT mRNA. The modified mRNA substantively reduced the excessive inflammatory response, and improved translation (17). Thus, the fundamental research to determine how cells sense pathogens, informed a use-inspired search for modified forms of mRNA that would escape detection of the PRRs. In addition, there was another development not as widely hailed but also critical to generating a safe RNA therapeutic. Recognition that there were trace contaminants of IVT reactions (18) led to specialized purification protocols that removed double stranded RNA, uncapped RNA, and short abortive RNAs that further reduced the unwanted immunogenicity of IVT mRNAs. The importance of the purification methods to generating a product that could be used for research in mammalian cells, and ultimately applied therapeutically in humans cannot be overstated, and is an excellent example of the importance of the Innominate quadrant.

Going forward, mRNA therapeutics will be further improved by the dynamic movement among the quadrants. Next generation RNA drugs will build on discoveries related to natural mechanisms that increase mRNA longevity. For example, one approach to significantly improve mRNA drugs is to circularize the mRNA (circRNA) so that it is resistant to degradation by exonucleases. The possibility of using circRNAs with an incorporated internal ribosome entry site (IRES) as a durable protein expression system was known for decades (19). However, only recently was it made more practical for generating therapeutic mRNA when an efficient method was developed to generate circRNAs of sufficient size to hold the open reading frame of a desired protein (20). Furthermore, greater expression of protein can be attained using self-amplifying RNA therapeutics (21, 22). The development of these RNA therapeutics derives in part from a fundamental understanding of the RNA biology (of the Semliki Forest Virus and Venezuelan equine encephalitis virus). However, the practical application of this knowledge has been advanced by separating the RNA dependent RNA polymerase function, from the open reading frame of the desired protein, into two constructs that represent a trans-amplifying RNA product more amenable to current manufacturing processes (21, 22). Thus, the promise of mRNA therapeutics will derive from a skillful navigation through all quadrants.

## Future at the Nexus

### A fresh opportunity: ARPA-H and the reimagined quadrants

#### A new strategy for success

ARPA-H, as recently announced, is poised to operate at the very nexus of science, engineering and medicine (23). As a DARPA-like agency focusing on health problems, ARPA-H and its anticipated success is predicated on the faithful implementation of the DARPA operative paradigm. DARPA is renowned for operating with a unique culture of risk-taking, failure-embracing, and rapid decision-making, where ambitious interdisciplinary teams that bring fresh ideas are continuously cycled over short periods. DARPA managers are granted high levels of authority and autonomy to move decisively. The NIH had been originally proposed as the location of ARPA-H to facilitate a health research and development focus. Recently, this logic led to the announcement of placement of this semiautonomous agency at the NIH with the Director reporting to the HHS Secretary (24).

Like DARPA, the NIH in its domain also has been tremendously successful in supporting the highest quality biomedical research in the Nation to achieve its mission. This organization, founded over 120 years ago, has a strong and established culture that values innovation and scientific inquiry, but differentially uses the time-tested process of two-stage scientific peer-review to identify and prioritize research investments.

It is critical to recognize that successful implementation of the DARPA strategy at ARPA-H will require substantially different yet complementary thought and execution processes. These must leverage what we have learned about the fluid nature of cognitive approaches that lead to transformational advances (25). Designating resources alone will not be sufficient for the new entity's success. Instead, modeling the DARPA culture of risk-taking, failing often and fast, facilitating a convergence of varied disciplines and approaches, and operating with a sense of urgency to deliver the target product in short timeframes will be required for success, but with a fluid interchange among this new agency and the brain trust of the NIH community. In so doing, appreciation of milestones in each quadrant as part of the journey to transformative innovation should be appreciated. A dynamic interaction among the quadrants will accelerate transformational advances.  This conceptual framework necessitates establishing a different mission-specific culture for this new agency, rather than redirecting an existing part of an historic institution. Yet, its linkage to the NIH with its strong history of discovery will allow the dynamic interchange to be most effective moving among the quadrants enroute to transformations. This speaks to the need to set up a new culture, which is far more practical and likely to be successful than the adaptation of an existing well-established culture at either agency.

#### The distinct advantage of the NIH location

Ensuring close collaboration among ARPA-H and the larger NIH will be beneficial to both. The colocation can help accelerate innovation and technology development by facilitating access to and leveraging key existing knowledge, resources, and fundamental discoveries that occur continuously at the NIH. Utilizing established relationships with other governmental and international agencies will be critical in the successful implementation and commercialization of any breakthrough technology. The reimagined, yet history-informed perspective of the future suggests that use-inspired basic research will surely lead to envisioned creations and some that are not yet conceived (just as Pasteur's observation of the behavior of cholera bacteria led to the development of vaccines) (26). Still a key lesson, as we have described, is that innovation flourishes in environments where frequent exchange across research domains, risk taking, and time pressure, are part of the operative approach. The synergy between the established NIH and the new entity ARPA-H should help maximize success for both. In this way, the collective community will be operating in this type of reimagined and more dynamic model, facilitating interactions, exchanges across quadrants to facilitate discovery, use inspired research and transformative application, as both serendipity provides and needs dictate.

#### A new opportunity for a long-held goal—health equity

A compelling opportunity that ARPA-H has, with this dynamic approach to transformation, is to accelerate equity in healthcare delivery. This warrants particular attention. It is a consequence of both the glaring need, and the observation that technological innovation has not purposefully targeted this problem at scale. As illuminated by the pandemic, health inequities in the United States are dramatic and increasing. The grand challenge of equity in delivering health to all people, a systemic problem, now needs a more holistic solution inclusive of technological innovation that expressly targets this issue (27). In addition to the study and use of genes, molecules, and cells, consideration of to whom, where and how innovations will be delivered must also be a design element. This is necessary to achieve effectiveness at the population level (27).

For example, technologies that diagnose and treat in one visit could help in settings where return trips are often difficult. Point-of-care diagnostics paired with a self-administered single-dose vaccine requiring no refrigeration would help achieve equity in the next epidemic. Early in-home detectors of disease that alert a person, identifies, and connects a suitable healthcare provider to them while also providing transparent person-specific cost coverage information is another. In complicated illnesses, like cancer and cardiovascular disease, surveillance targets and analytics could reduce genetic risks with timely monitoring and informed treatment. These could provide actionable feedback to patient and physician. Such dynamic and action-oriented information could make a big difference at the population level and in communities, where regular medical contact is not typical. ARPA-H would offer the opportunity to develop and support the deployment of technologies to affect this new approach that has been previously envisioned but has not been implemented (27, 28).

At ARPA-H, proposals for funding and research management could be required to include applied research (Edison's quadrant) and commercialization steps for public use (Innominate quadrant), possibly with industry collaborators as  components. The new organization could pursue technologies that are health equity facilitating as part of its mandate to ensure benefits accrue to all. The functional synergy of two powerful and time-tested agencies through this new one could create brilliant breakthroughs in health. The driving vision of global healthy longevity could be facilitated through what would metaphorically be a new orbital at the NIH. This would be a tremendous advance (29).

### Training the next generations: the convergents

#### Educating those who will trailblaze 22nd century healthcare

New schools that integrate medicine and engineering, based on the convergence concept, have been created to purposefully train individuals to become creative problem solvers and team science members with a unique understanding of both the problem and solution spaces.

The Carle Illinois College of Medicine at the University of Illinois is the first engineering-based medical school. The curriculum is built at the intersection of engineering and medicine, with most courses designed by a team composed of a basic scientist, clinician, and engineer. Students earn an MD in 4 years. Similarly, but with some distinctive features that are also a first, the newly created School of Engineering Medicine (EnMed) at Texas A&M in collaboration with the Houston Methodist seeks to train a new type of healthcare professional that is a blended hybrid of a Physician and Engineer, called a *Physicianeer*. These individuals will be purposefully trained to be both consummate medical doctors and to innovate solutions to medical problems. Their work will derive from an improved fundamental understanding of health and disease, leading to use-inspired inventions. And as history has taught us, one can reasonably expect that such innovations will in turn advance our fundamental understanding of health and disease, generating more discoveries. Students at EnMed, through a unique blended curriculum, earn two degrees in 4 years, an MD and a Master of Engineering Innovation. To anchor the goal of training problem solvers, students are required to have an invention that addresses a healthcare challenge. Thus, the Vision statement: “EnMed graduates will uniquely help to transform healthcare as Physicianeers. This will be achieved through convergence born innovations that improve the *understanding and treatment* of disease.”

A third established program that focuses on related training, but from a different educational starting point, is the Medical Innovators Development Program at Vanderbilt. This program trains individuals with an existing PhD in basic or applied sciences in a 4-year, innovation-centered MD curriculum. As such, it has those unique features. Physician innovators are developed by bringing together engineers and applied scientists from diverse backgrounds into a clinical environment. Still a fourth emerging and related initiative is the Center for Engineering and Precision Medicine, recently announced as a joint effort by Rensselaer Polytechnic Institute and the Icahn School of Medicine at Mount Sinai. This proposes a suite of PhD and MS degrees with research and education focused on technological innovation to advance healthcare. Other related programs include Columbia's Rising Stars in Engineering in Health for those transitioning to faculty positions, and Yale's recent engineering surgery-directed program, Master of Science in Personalized Medicine and Applied Engineering.

To facilitate convergence in all of these programs, it is important to train the next generation in the four “T”s of transdisciplinary, team-based, translational, and transcultural orientation. This orientation is inclusive of a set of values, attitudes, beliefs, behaviors, and analytical approaches which promote interdisciplinary collaborations. Such training will facilitate dynamic movement among all quadrants.

The overarching vision and aspiration, achieved through a dynamic interplay among the areas of fundamental discovery, application, and use-inspired basic research, is Trailblazing 22nd Century Medicine to benefit all (27, 28). In this way, by educating generations of students who think at the nexus of science, engineering, and medicine, we can improve the lives of humankind in our contemporary society. We can also advance the art and science of innovation to the benefit of all generations to follow.

## Conclusion

In sum, this Perspective seeks to accelerate a deeper integration of Science, Engineering, and Medicine among academic, government, and industrial communities. In this piece, we emphasized the more explicit consideration of the time domain in the execution of research where these fields are dynamically converged across pure basic research, applied research, and use-inspired basic research domains, supported by advances in catalytic technologies and platforms. The emphasis is on appreciating and supporting a fluid interchange among these quadrants over time to continuously yield transformative innovations. Historically, funding agencies, for example, have not generally encouraged, or embraced this dynamic in their initiatives. Major advances in human well-being can be derived from doing so.

## Authors’ Contributions

Both authors contributed to the concepts and illustrative examples, composition and editing of the manuscript.

## Acknowledgments

This work is supported in part by the Welch Foundation funding and the Texas Governors University Research Initiative award to R.I.P. and grants to J.P.C. (NIH R01s HL133254, HL157790, and HL148338; the Cancer Prevention and Research Institute of Texas [RP150611 and RP200619]; the George and Angelina Kostas Research Center for Cardiovascular Medicine; and the Robert J. Kleberg, Jr and Helen C. Kleberg Foundation). The authors greatly benefitted from thoughtful and provocative discussions with Richard Ehman, MD, Mayo Clinic; Gang Bao, PhD, Rice University; Daniel Kiss PhD, Houston Methodist Hospital; Bruce Rosen, MD, PhD, Harvard University; Michael Paolini, MD and Rhome Hughes, MD, Texas A & M University; and Norbert Pelc, PhD, Stanford University.
